# Supplementary figures and images for: Bird song comparison using deep learning trained from avian perceptual judgments
Source: PLoS Comput Biol. 2024 Aug 7;20(8):e1012329. doi: 10.1371/journal.pcbi.1012329 (PMC11333001; doi:10.1371/journal.pcbi.1012329)

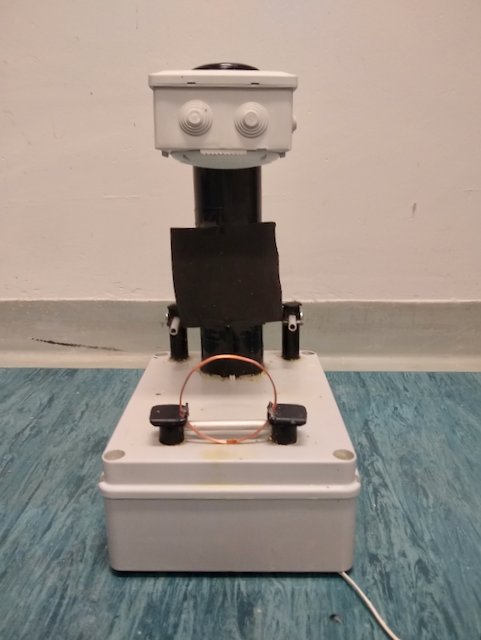

Supplement: S1 Fig — For a schematic image see Fig 1. (TIFF) [file pcbi.1012329.s001.tiff]

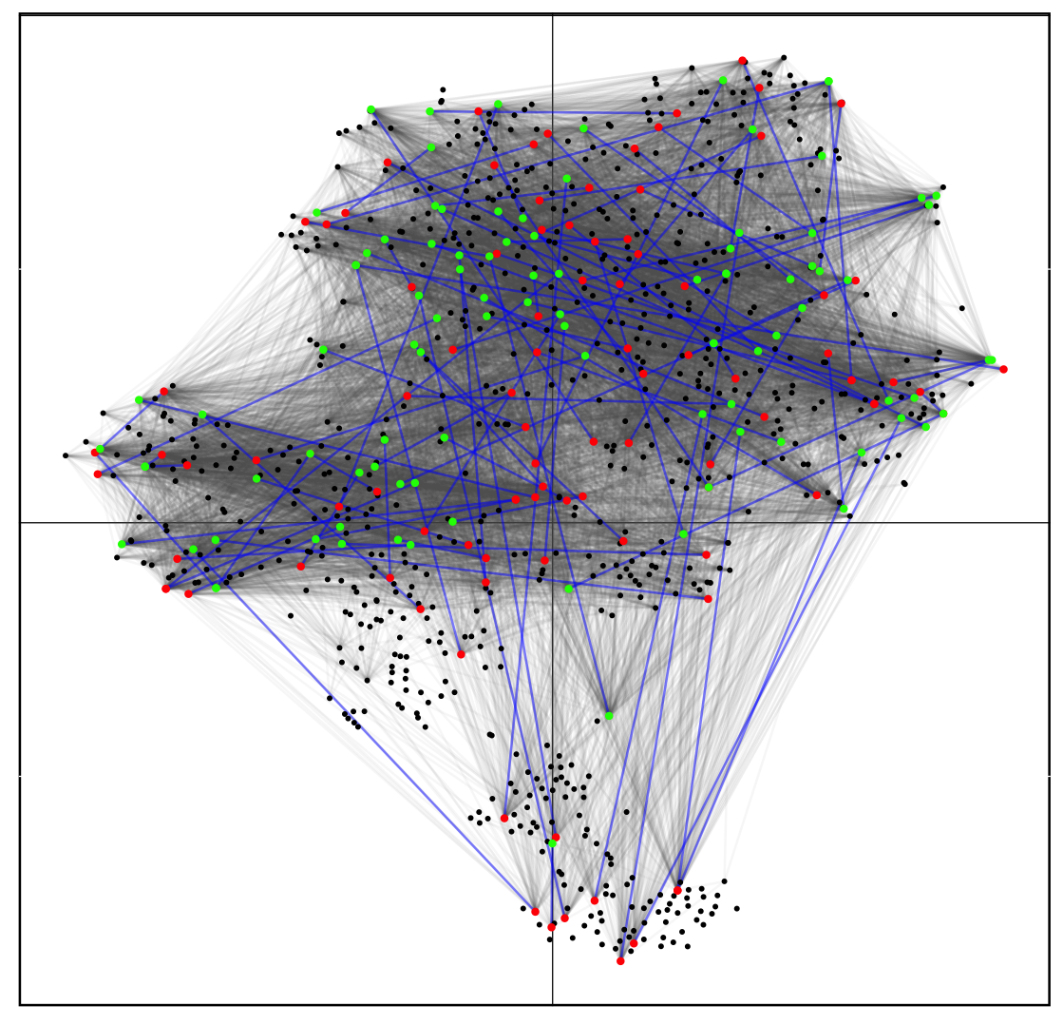

Supplement: S2 Fig — Extracted from 64-dimensional embedding using T-SNE for the projection with Euclidean distances. Sets of training stimuli are connected with blue lines, connections between probes and training stimuli with grey lines. Stimuli used for training on the left side are coloured in red, training stimuli on the right side are coloured in green. (TIFF) [file pcbi.1012329.s002.tiff]

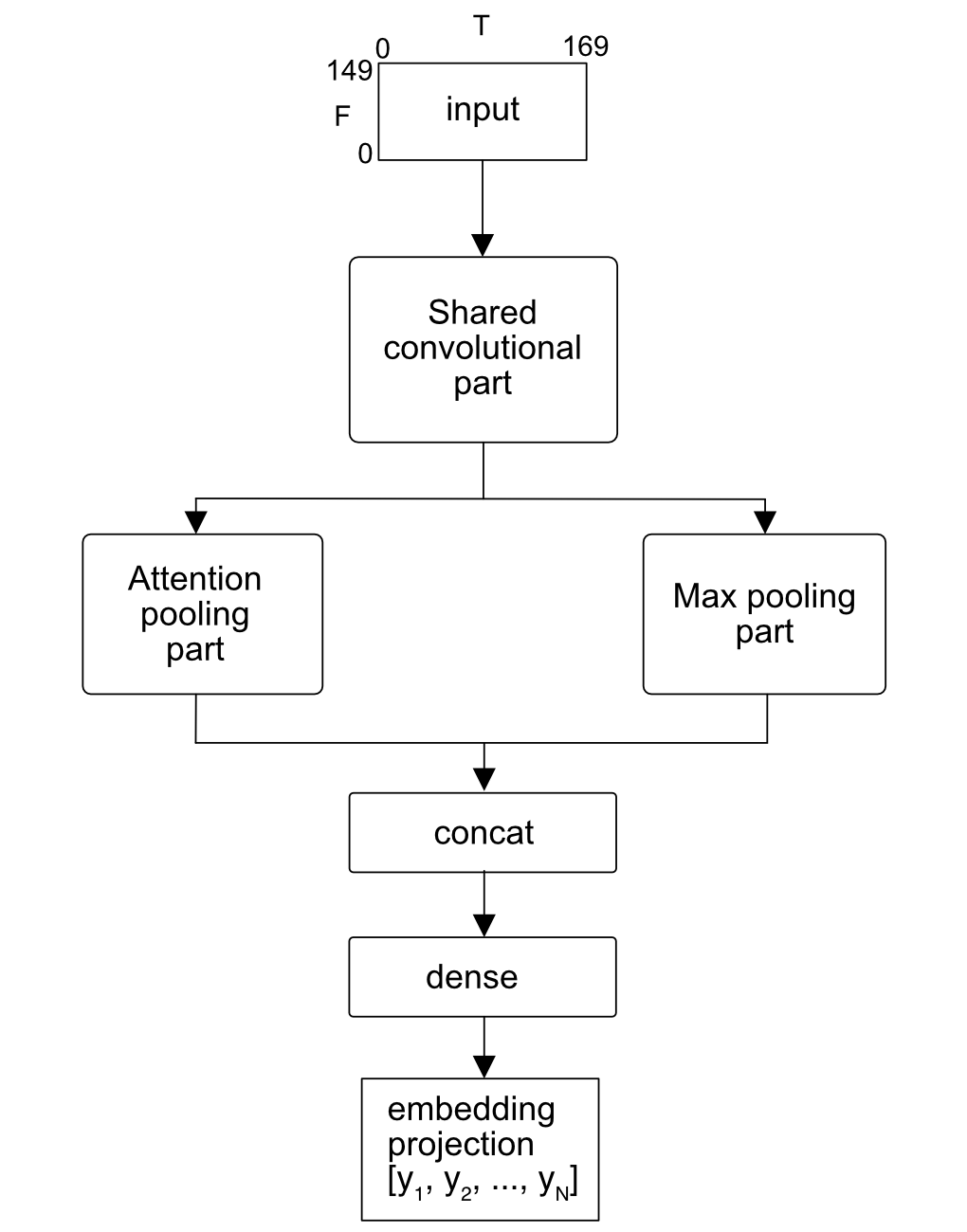

Supplement: S3 Fig — Input is a time-frequency representation of a recording, with shape 170 time frames and 150 frequency bins. The shared convolutional part performs a number of convolutions, batch normalisations, and leaky-ReLU non-linearities to the input. The output of the convolutional part is used as input to two different branches of the network, one performing attention pooling and the other performing max pooling. The results of the two branches are concatenated together and used as input to the final layer of the network; a dense layer that performs projection of the input into an N-dimensional embedding space. Figure adapted from [20]. (TIFF) [file pcbi.1012329.s003.tiff]

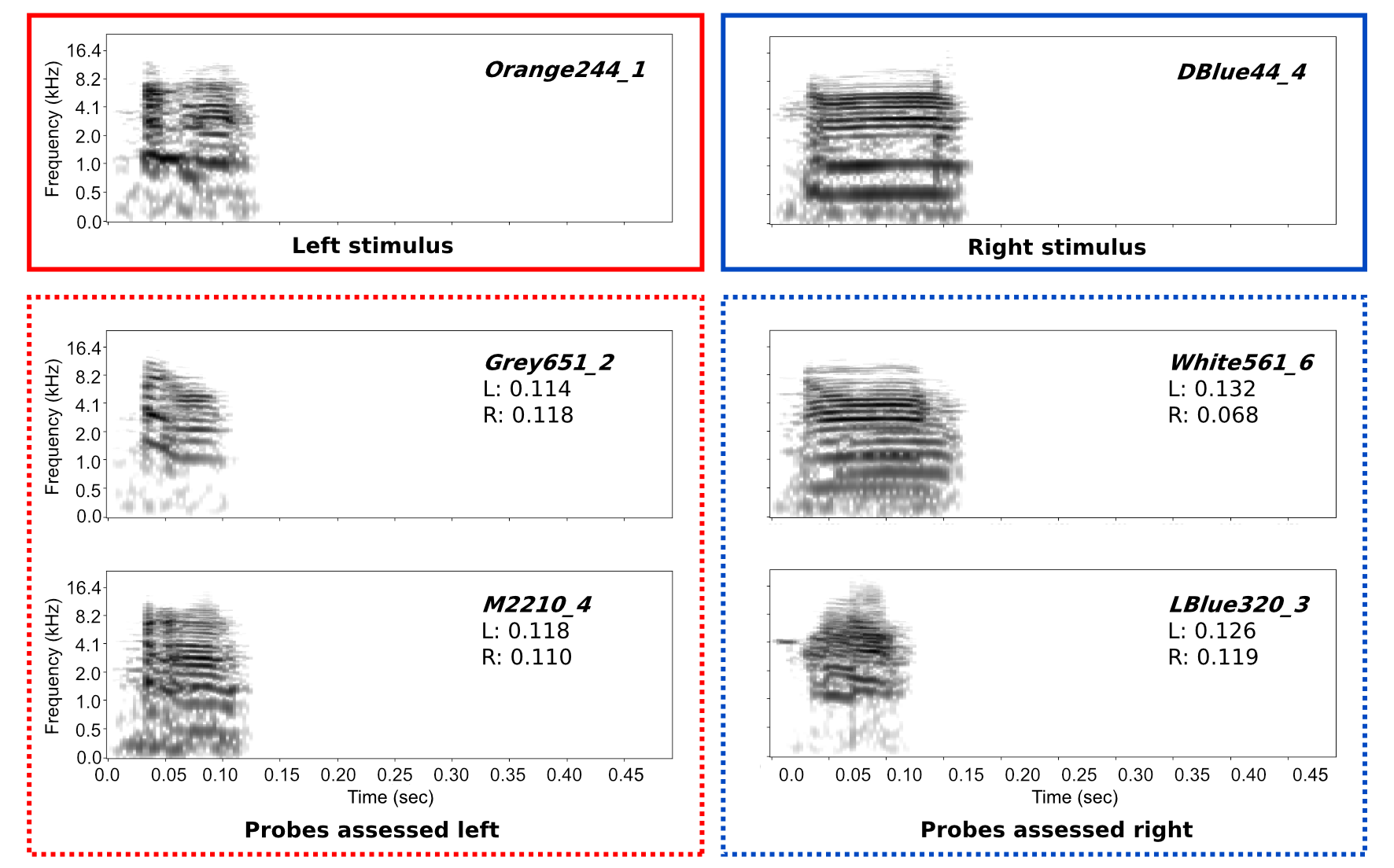

Supplement: S4 Fig — Probes in the left column were assessed by the bird as being more similar to the left stimulus, and probes in the right column were assessed as being more similar to the right stimulus. Numbers indicate the dissimilarity between the probe and the left (L) and right (R) stimulus according to Luscinia. Note that for 3 probes the bird agrees with the assessment of Luscinia, while for one probe (M2210_4) it disagrees with the Luscinia assessment. (TIFF) [file pcbi.1012329.s004.tiff]

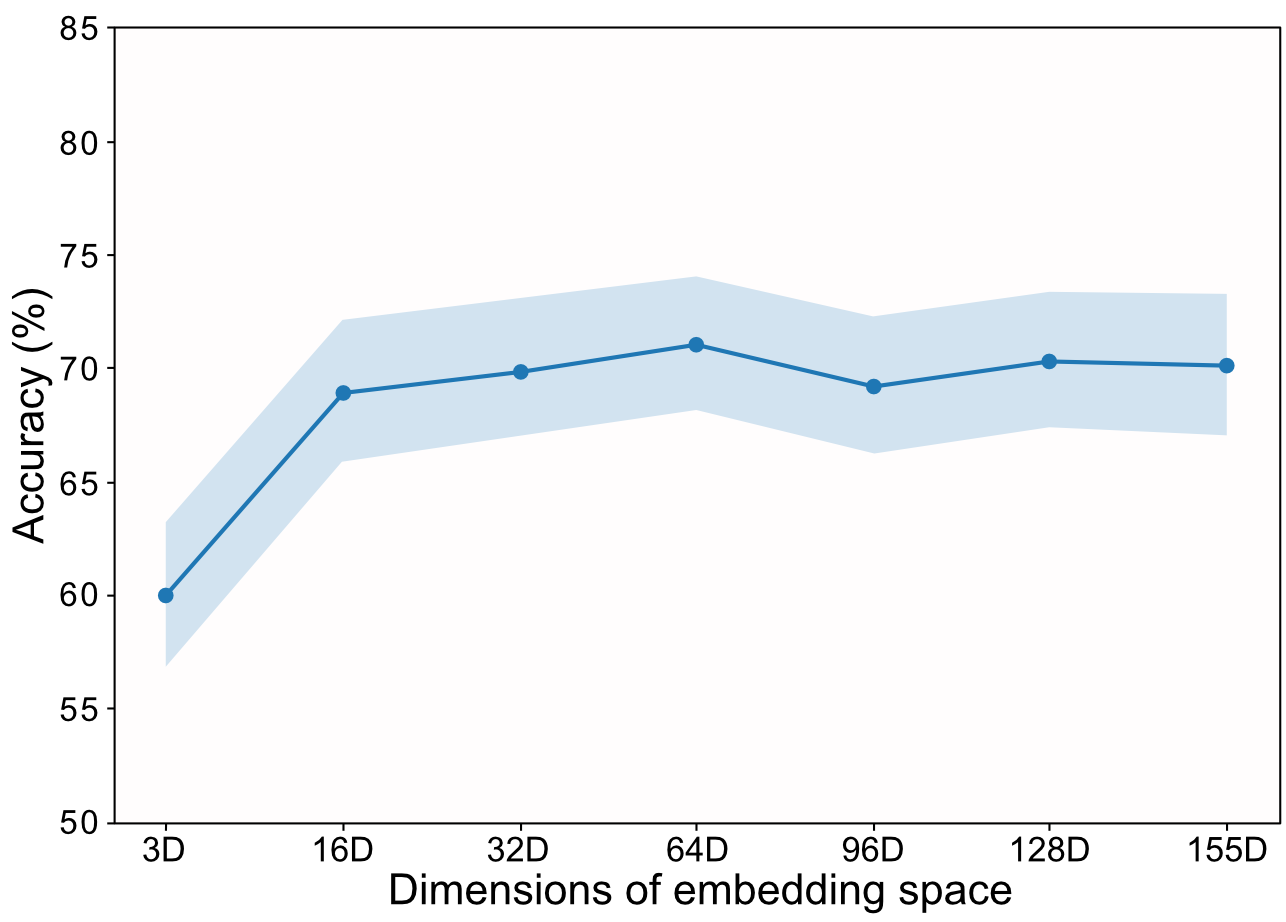

Supplement: S5 Fig — (TIFF) [file pcbi.1012329.s005.tiff]

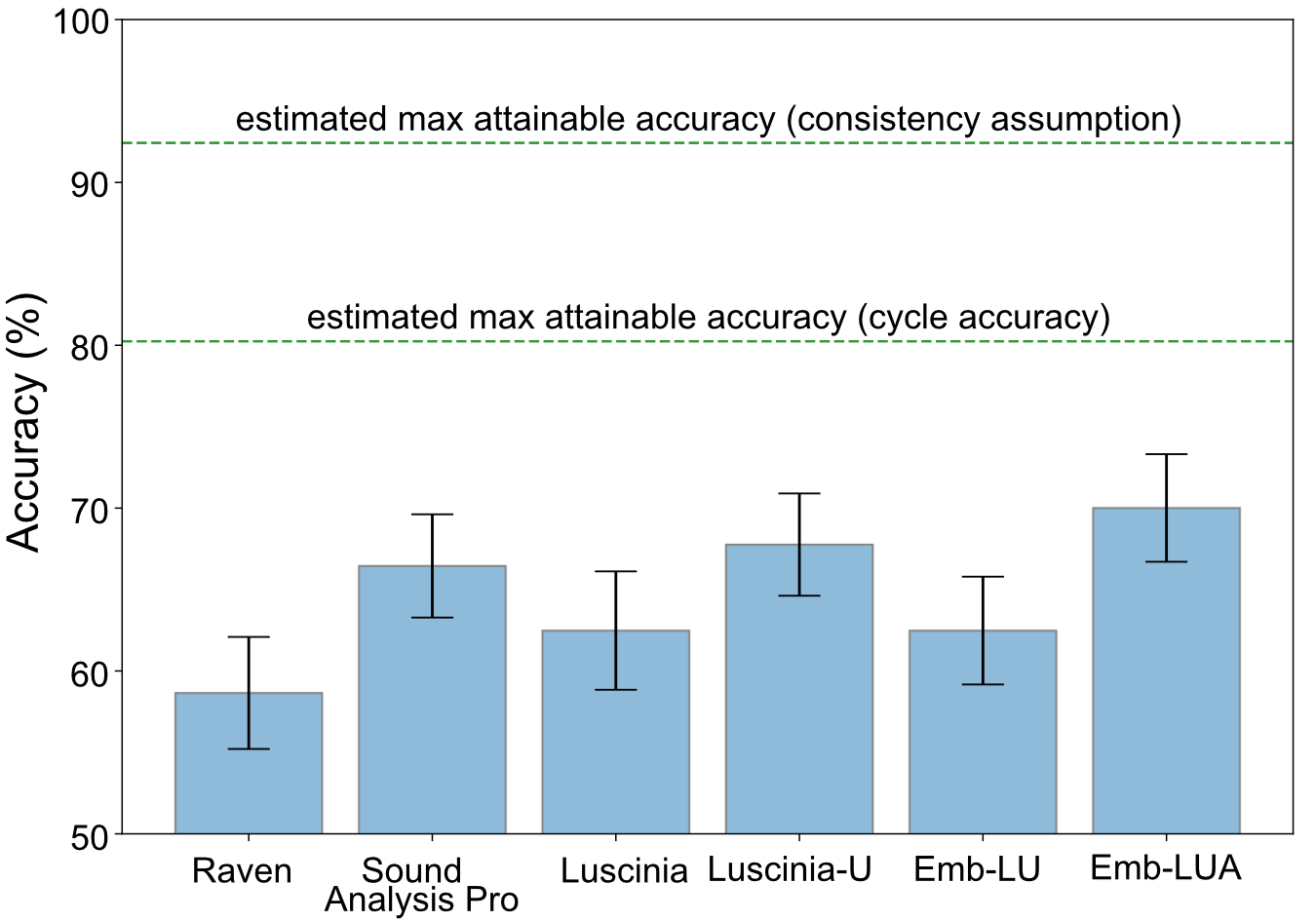

Supplement: S6 Fig — Details are the same as in Fig 5. (TIFF) [file pcbi.1012329.s006.tiff]

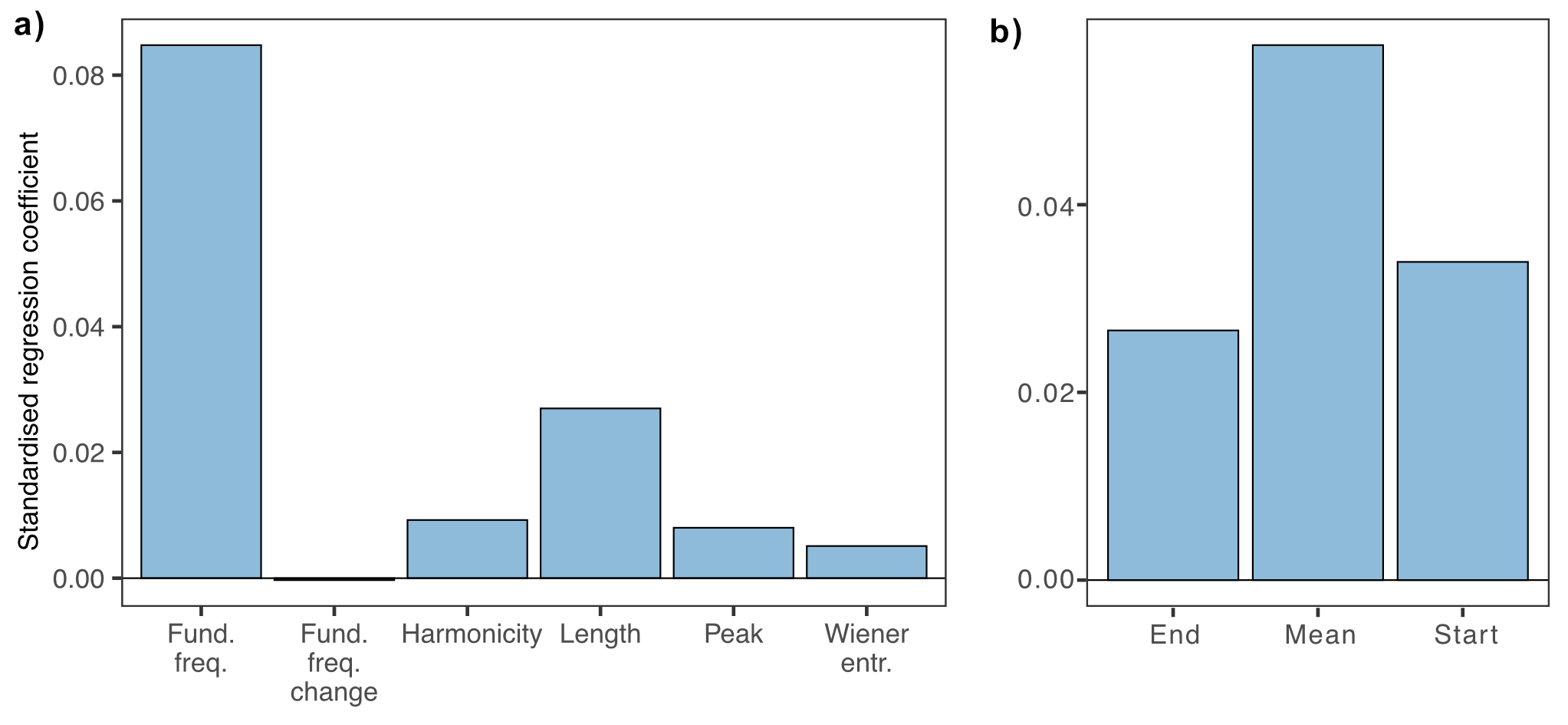

Supplement: S7 Fig — Results show the standardised regression coefficients (A) when grouping the variables by the 5 overall song features and syllable length and (B) when grouping the variables by summary statistics start, end and mean. Each bar represents the standardised regression coefficient from the respective MRM analysis. (TIFF) [file pcbi.1012329.s007.tiff]

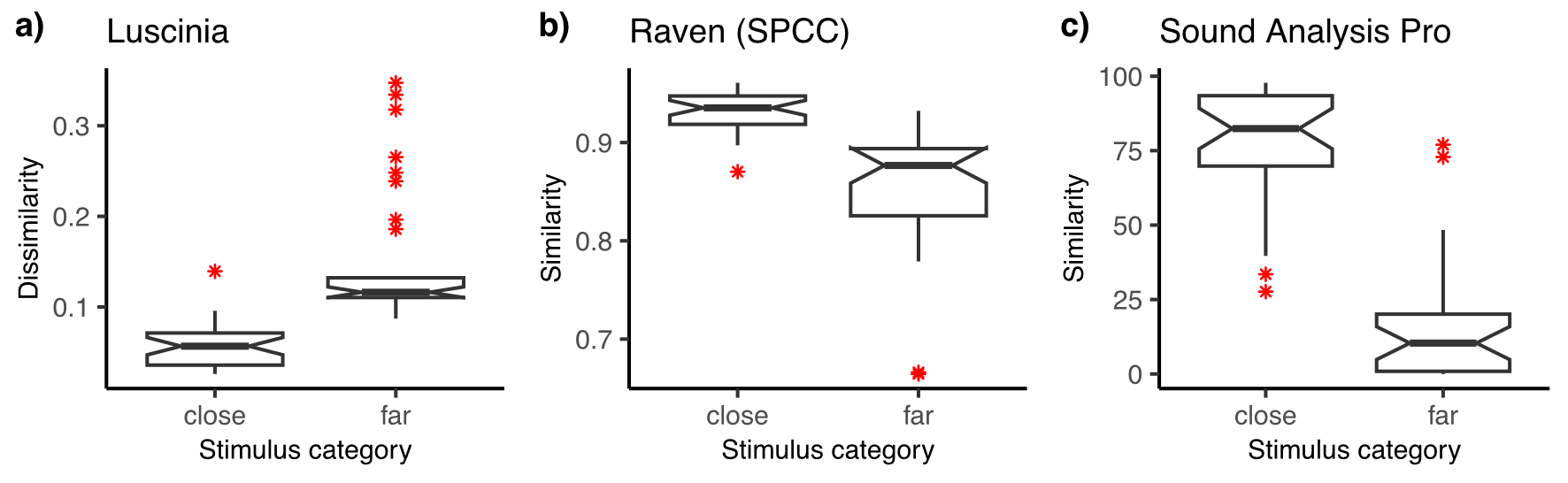

Supplement: S8 Fig — Comparison of similarity within a stimulus category (close: core stimulus exemplar in group ‘A’ with the 7 other syllables of group A and the same for group B) and between stimulus categories (far: between core stimulus exemplar A and the 8 stimuli in group B, and vice versa) for Luscinia, SAP and Raven (SPCC) for the evaluation set. For all three algorithms the close and far groups separate out, showing a higher similarity for the close group than for the far group. (TIFF) [file pcbi.1012329.s008.tiff]
