# Supplementary material for: Bird song comparison using deep learning trained from avian perceptual judgments
Source: PLoS Comput Biol. 2024 Aug 7;20(8):e1012329. doi: 10.1371/journal.pcbi.1012329 (PMC11333001; doi:10.1371/journal.pcbi.1012329)
Supplement: S1 Table — (PDF) [file pcbi.1012329.s009.pdf]

Table S1: Table of parameter settings for Luscinia.

Parameter	Weighting prior to training	Weighting post-training
Mean frequency	0.0925	0.0568
Peak frequency	0.0925	0.0273
Fundamental frequency	0.0925	0.3788
Peak frequency change	0.0925	0.0122
Fundamental frequency change	0.0925	0.0597
Normalized fundamental frequency	0.0925	0.2369
Wiener entropy	0.0925	0.00021
Harmonicity	0.0925	0.0832
Time	0.25	0.0443

The table shows the parameter weightings in Luscinia set before training, and achieved after training. Luscinia's DTW algorithm aligns acoustic features as they vary over the length of the syllable. Different features can be weighted more or less heavily.
