# Supplementary material for: Bird song comparison using deep learning trained from avian perceptual judgments
Source: PLoS Comput Biol. 2024 Aug 7;20(8):e1012329. doi: 10.1371/journal.pcbi.1012329 (PMC11333001; doi:10.1371/journal.pcbi.1012329)
Supplement: S2 Table — (PDF) [file pcbi.1012329.s010.pdf]

Table S2: Table of MRM standardised regression coefficients.

Parameter	Standardised regression coefficient	p-value
Length	0.0191	0.0001
Fundamental frequency mean	0.0781	0.0001
Fundamental frequency start	0.0566	0.0001
Fundamental frequency end	0.0203	0.0298
Fundamental frequency min	0.0387	0.0001
Fundamental frequency max	-0.0284	0.0001
Frequency change mean	-0.0014	0.7685
Frequency change start	0.0014	0.7185
Frequency change end	-0.0023	0.523
Frequency change min	-0.0002	0.944
Frequency change max	0.0013	0.7018
Harmonicity mean	-0.0027	0.3887
Harmonicity start	0.0020	0.4994
Harmonicity end	0.0085	0.0035
Harmonicity min	0.0155	0.0006
Harmonicity max	-0.0138	0.0002
Wiener entropy mean	0.0108	0.0074
Wiener entropy start	-0.0029	0.3528
Wiener entropy end	-0.0070	0.0315
Wiener entropy min	0.0052	0.1622
Wiener entropy max	0.0044	0.171
Peak frequency mean	0.0083	0.0108
Peak frequency start	-0.0023	0.4209
Peak frequency end	-0.0007	0.8319
Peak frequency min	0.0109	0.001
Peak frequency max	-0.0031	0.2632

The table shows the result of the MRM analysis on all 25 acoustic features and syllable length. The model explained 68% of the variation in embedding scores.
