# Supplementary material for: Bird song comparison using deep learning trained from avian perceptual judgments
Source: PLoS Comput Biol. 2024 Aug 7;20(8):e1012329. doi: 10.1371/journal.pcbi.1012329 (PMC11333001; doi:10.1371/journal.pcbi.1012329)
Supplement: Attachment — Submitted filename: PCOMPBIOL-D-23-01544_reviewer.docx [file pcbi.1012329.s011.docx]

Main comments:

In this article the authors aimed at constructing a neural network that emulate zebra finches responses to an auditory task of discrimination of song syllables. More precisely their aim is to obtain an embedding that reflects how similar song syllables are by zebra finches standards. Such an algorithm would be really useful in the fields of animal behavior and neuroscience that investigate how songbirds learn to imitate songs. In these fields, it is assumed that the similarity as measured by human perception biased algorithms is the error signal that birds use to guide their performance in vocal imitation. The obtention of a neural network that emulates bird ground truth auditory perception of song similarity would definitely be a game changer in the birdsong field. To achieve their aim the authors constituted an impressive dataset of birds behavioral responses to an auditory task of song syllable classification and leveraged a network architecture with its learning rules that was previously developed for human behavioral rating of textures.

However, I have some reservations regarding the protocol with which the behavioral data used as training and testing sets to the neural network were obtained. In particular, I am not convinced that the behavioral responses obtained from the birds were totally devoid of any human-perception based algorithm bias. Indeed, in the auditory classification task that the birds are asked to perform in order to measure their perception of syllable similarity, birds are first trained to classify two sets of syllables before the introduction of probes that test the birds perception of similarity of these probes to the two training sets. Each training set consists in 8 syllables that were chosen such as to be adjacent to each other in the acoustic space defined by Luscinia. I am concerned that such an approach is actually teaching the birds Luscinia’s acoustic similarity rules and that their responses are actually biased to respect Luscinia’s sense of what should be considered as similar or dissimilar. Note that among the 99 set pairs of stimuli on which the birds were trained, only half (46) were correctly classified by birds, indicating that while the authors were attempting to ensure that sets were sufficiently distinct to be discriminated by birds, based on Luscinia’s rank metric of dissimilarity, the task was in fact not easy for birds. In my opinion this point to the fact that Luscinia’s ranking metric of syllable similarity is far from bird perception and the experimental design force the birds to use a human perceptual biased rule to classify song syllables as similar or dissimilar. This effect is then also visible in the comparison of the network performance at emulating bird responses, with that of other popular acoustic softwares (Raven, Sound Analysis Pro and Luscinia) in figure 3. Surprisingly (or maybe not!), Luscinia is the software that shows the highest performance as compared to the other softwares. If Sound Analysis Pro (SAP) had been used to decide of the stimulus sets, would SAP similarity measures had been the best software to emulate birds behavior and would the embedding of the deep network show performance more similar to that of SAP?

I would be convinced that the embedding obtained really constitute an unbiased perception space of zebra finches song syllables if another algorithm was used to decide of the sets of song syllables and birds’ perceptual space (network embedding) obtained with these data remained unchanged to the previous embedding.

Overall there are many missing details in how the data analyses were performed. I tried to flag that throughout the article with my line by line comments. Critical points are the justification of the choice for ranking syllables according to acoustic similarity instead of using a more linear metric (why not use directly the dissimilarity measure? mean rank could mean very different thing depending on where you are in the acoustic space) and how bird individual variability is taken into account throughout the analyses.

Furthermore, I think the paper would be strengthen by a thorough investigation of which acoustic features are important by zebra finch standards in the evaluation of syllable similarity. Revealing the perceptual map of birds would be a very interesting biological result.

Line by line comments

Line 50 « DTW » shouldn’t be « Luscinia »?

138: which similarity measures were chosen? What heuristic is chosen for the ranking of syllables?

What prevent another bird from interfering with a given subject behavior when activating the operant feeder? Providing a picture of the design would give a better idea of the design.

In figure S1, consider color coding the 2 sets of training stimuli A and B in the projection for clarity.

What was the average distance of within category (7 most similar syllable to A) vs between categories (median(A) to median(B))?

Consider adding a figure that shows example spectrograms of one A-B set and a few probes with their Luscinia distances along with bird classification.

171: does 70% controls for bird ID = all birds have to be at 70% to move forward with probes?

How often do bird not take decision (no perch hope/exit of the feeder)? How was such behavior quantified? Would it be counted as a wrong response?

175: what is the similarity metric used by Luscinia?

175: on basis **of** their similarity

176: the rank of ~~the~~ each probe stimulus

176: according to previous paragraph two sets of 8 stimuli are used with the probes. Here, is the initial (presumably close to the median?) stimulus of each set chosen as a reference for the triplet? How do you take into account the difference of variance within each set (e.g. differences across the 8 A stimuli)?

177-179: rephrase this sentence because the sign of the difference totally depends on whether you are doing L-R or R-L, better indicate that the probe is more similar to the stimulus for which it has the smallest rank.

180: over which stimuli is the mean rank difference calculated?

Consider adding mathematics formula to increase the transparency of calculations here.

How many responses by the same bird to the same probe are obtained before assessing bird’s assessment? Do you merge all birds responses together?

What is a cycle of accuracy? How do you define a test period?

192-194: nonsense, revise sentence.

222: what is the time window or bin size chosen for the mel spectrogram?

234-235: what is defined as ambiguous and unambiguous behavioral data ?

241: denotes the squared ~~the~~ Euclidean…

177/240: it is somewhat confusing to chose a negative distance of rank to denote stimuli that are closer in the acoustic space according to Lusciana and then define positive stimuli (samples) for a given anchor as being closer than negative stimuli.

242 and equation: define what N is (I assume total number of anchors = probes). A single triplet should only be a set of 3 stimuli and not the ensemble of triplets? According to the sentence, the Loss function is given for a single triplet, but the equation is giving it over all triplets in the dataset (sum over N?).

250: define cycle accuracy, explain what time binning is used to calculate it, what is the rolling time period?

It’s an important point that scrounging is made possible in this design. An estimate of how often it happens would be important to develop here and present earlier when presenting the operant feeder.

What do you mean by “side decisions” do you mean systematic “errors”?

261: what is a cycle?

262: What was the representation of each syllable in these 2099 triplets? In other words in how many triplets of average was a given syllable being an anchor? Are some syllables found to be anchors more often than others?

273-274: how is side consistency calculated, over how many number of responses to the same stimulus? Is it correct to assume like as on line 260-261 it is calculated for each bird? Perception choices could be highly dependent on individuals, how is that accounted for?

Revise equation1 it is confusing with respect to the subsequent equations. Be consistent in the notations. Either D is the squared Euclidean distance or the Euclidean distance but not both.

279-287: I am a bit confused by your new loss function in equation (4) as compared to (1). First the use of an exponential on the difference of distance for unambiguous tripets makes it highly susceptible to outliers: a single unambiguous triplet with a big difference of distance (pu>0) would very rapidly decrease the Loss to very low values (exponentially low!).
Note that Kumari et al (2019) does not use 1-exp(pu) but exp(-pu) for the unambiguous triplet. Please address why you are doing differently.

Second, can you give an intuition for why adding a penalty to the difference of distance of ambiguous triplets is increasing the performance of distance prediction for unambiguous data?

296: what do you call “Luscinia-U decisions in training”? Do you mean the ranking obtained through Luscinia acoustic measures of similarities? I see that this explain below. It is probably worth pointing to it or giving a short explanation here.

300-305: please spell out/give an equation for what you define as cycle accuracy, idem for the upper bound (self-rater agreement? Inter-rate agreement? How do you estimate these values?).

Table 1: indicate how birds decisions are used in models checked for U and A: training + testing? How many triplets were constituting the Luscinia-U dataset (in other words by how much the bird dataset was augmented by these “machine” data?)?

320: can you indicate how sound features are weighted in the original version of Luscinia for the calculation of the dissimilarity value, which I believe is the version used to rank syllables and choose the initial triplets with which birds were trained and tested? Note that it would be useful to describe this dissimilarity measure as a stand-alone paragraph earlier because it is used to decide of the behavioral sets of stimuli. Then the improvements in Luscinia U can go in a subsequent paragraph.

321: “same training data that was used to train neural networks” -> according to table 1 there are several versions of these data, can you be more specific? I assume you are using the behavioral data only, but then all triplets? Or only unambiguous triplets?

322: how was the likelihood calculated for each triplet? The DTW algorithm described gives a single dissimilarity measure for each pair of stimuli, how did you convert these differences of distances into probability and loglikelihood?

323: avoid jargon, “burn-in”

326: what was the rule in the MCMC approach to update or keep weights used in the DTW algorithm?

335: dep->deep

334: by “additional AXB triplet decisions” do you mean that you are adding triplets that have not been investigated by birds or that you are giving new responses to triplets for which you already obtained birds responses?

Figure S2: The schematic does not indicate how the bird behavioral responses are used to train the model. From the description in the paper we are expecting to see a model that predict the distances Dap and Dan from 3 sounds and this is not what is offered to the reader in this figure (well, the input seems to represent one spectrogram but we would expect three!). Then it would be useful to understand where the embedding projection is situated in the network (one but last layer?) relative to the actual final output which should be two values of distances.

Then what measure of distance are you computing from the embedding projections of two new input sounds to compare with other software dissimilarity value metrics?

350: an multiple -> a multiple

353: was the Euclidean distance for each acoustic feature calculated with a weight of 1 for each of the 5 measurements (mean, max, min, start and end values) ?

362: given that the system was supposed to deliver 10-15% probe stimuli, how it comes that only 25,999 out of 1,116,214 trials were probe trials? Are you including in the count training phases (steps 1 to 4c?)

363: What does a testing period correspond to? how long were the testing periods? What was the decision criteria to switch from one period to the next?

365: can you speculate why only half of the sets were discriminated above chance by the birds? Is there any correlation with the average acoustic distances (as evaluated by Luscinia?) within sets? Is the mean +/- SE the average value given over birds and the 46 sets? What does the SE represents here? Variability over birds and sets or birds only or sets only?

Please provide a clear distribution of the cycle accuracy for all stim sets and all birds.

Some more statistics of the dataset are missing to fully appreciate how representative the birds responses are. For instance: Were all 99 stimulus sets evaluated by all 23 birds? How many responses were obtained by a given bird for a given probe?

369-371: I have a very hard time to understand what is represented in Figure 2 and how you are reporting the birds performance in the text. What is the standardized density of choices? Can you represent the birds’ response variability? Can you explain why the birds behavior is not symmetric with respect to zero? For some reason there seem to be a bias where distance to left stimuli needs to be smaller for being correctly classified as left stimuli as compared to right stimuli. Can you explain the peak at -0.2 for both left and right lines?

Can you give any statistical test results for the comparison of model performances? How can you control for the different amount of data used?

382-387: the method section explaining how the MDS projection is obtained is missing (what did you use as input to the MDS?....).

The set of acoustic features used in Luscinia is not investigating temporal patterns of amplitude, that could account for the left over variations. Could you do a regression based on spectrogram distances to investigate what are the perceptual dimensions used by birds to perform this task? Alternatively it could be useful to investigate the embedding that best emulate birds behavior and interpret the maximum axis of variance: what are the birds paying attention to when they are doing this discrimination task on song syllables?

429-432: reference?

451-453: I don’t see in the result section where you compared the acoustic parameters weights in U-Luscinia and the Betas of the model. Moreover, according to Table S1 and Figure S5 these acoustic features don’t seem to be exactly the same.
