# Supplementary material for: Bird song comparison using deep learning trained from avian perceptual judgments
Source: PLoS Comput Biol. 2024 Aug 7;20(8):e1012329. doi: 10.1371/journal.pcbi.1012329 (PMC11333001; doi:10.1371/journal.pcbi.1012329)
Supplement: Attachment — Submitted filename: Response_reviewer_comments.docx [file pcbi.1012329.s012.docx]

**Reviewer #1:**

Main comments:

In this article the authors aimed at constructing a neural network that emulate zebra finches responses to an auditory task of discrimination of song syllables. More precisely their aim is to obtain an embedding that reflects how similar song syllables are by zebra finches standards. Such an algorithm would be really useful in the fields of animal behavior and neuroscience that investigate how songbirds learn to imitate songs. In these fields, it is assumed that the similarity as measured by human perception biased algorithms is the error signal that birds use to guide their performance in vocal imitation. The obtention of a neural network that emulates bird ground truth auditory perception of song similarity would definitely be a game changer in the birdsong field. To achieve their aim the authors constituted an impressive dataset of birds behavioral responses to an auditory task of song syllable classification and leveraged a network architecture with its learning rules that was previously developed for human behavioral rating of textures.

However, I have some reservations regarding the protocol with which the behavioral data used as training and testing sets to the neural network were obtained. In particular, I am not convinced that the behavioral responses obtained from the birds were totally devoid of any human-perception based algorithm bias. Indeed, in the auditory classification task that the birds are asked to perform in order to measure their perception of syllable similarity, birds are first trained to classify two sets of syllables before the introduction of probes that test the birds perception of similarity of these probes to the two training sets. Each training set consists in 8 syllables that were chosen such as to be adjacent to each other in the acoustic space defined by Luscinia. I am concerned that such an approach is actually teaching the birds Luscinia’s acoustic similarity rules and that their responses are actually biased to respect Luscinia’s sense of what should be considered as similar or dissimilar. Note that among the 99 set pairs of stimuli on which the birds were trained, only half (46) were correctly classified by birds, indicating that while the authors were attempting to ensure that sets were sufficiently distinct to be discriminated by birds, based on Luscinia’s rank metric of dissimilarity, the task was in fact not easy for birds.

In my opinion this point to the fact that Luscinia’s ranking metric of syllable similarity is far from bird perception and the experimental design force the birds to use a human perceptual biased rule to classify song syllables as similar or dissimilar. This effect is then also visible in the comparison of the network performance at emulating bird responses, with that of other popular acoustic softwares (Raven, Sound Analysis Pro and Luscinia) in figure 3. Surprisingly (or maybe not!), Luscinia is the software that shows the highest performance as compared to the other softwares. If Sound Analysis Pro (SAP) had been used to decide of the stimulus sets, would SAP similarity measures had been the best software to emulate birds behavior and would the embedding of the deep network show performance more similar to that of SAP?

- We have now also calculated the similarity within a stimulus category (close: core stimulus in group A with the 7 other syllables of group A and the same for group B) and between stimulus categories (far: between core stimulus A and the 8 stimuli in group B, and vice versa) for Luscinia, SAP and cross correlation for the test set. For all three algorithms the close and far groups separate out, showing a higher similarity for the close group than for the far group. Therefore, we claim the very strongly-separated stimulus categories (for this part of the experiment) are uncontroversial distinctions that all algorithms agree upon.

- Moreover, the acoustic similarity for the training phase was at such a coarse level that multiple algorithms agree on these distinctions (hence, by extension, we claim the same for natural acoustic judgments) and the fine differences do not come into play in the training phase.
- Although we think that we have some evidence that SAP doesn’t match bird decisions quite as well as Luscinia and the trained methods, this is far from our main point with this study.

I would be convinced that the embedding obtained really constitute an unbiased perception space of zebra finches song syllables if another algorithm was used to decide of the sets of song syllables and birds’ perceptual space (network embedding) obtained with these data remained unchanged to the previous embedding.

- Using another algorithm to decide the sets of song syllables would require running a new set of stimuli with the birds. Even if that had been designed in from the start, it would have reduced (e.g. halved) the sample size used to train the algorithm, and thus we judged it not feasible.
- The dataset with syllables consisted of songs from birds from a single population (different from the tested population) and from each bird we used several exemplars of its songs. Because of this there was quite a high amount of song sharing and a lot clustering in the syllable data. The syllables selected for each stimulus set were therefore syllables that were very similar to each other.
- Even with cross correlation, a relatively inaccurate method of comparison, the two stimulus sets are clearly different (see figures above). What we are aiming for is a situation where the training stimuli are tightly clustered and clearly different from each other. Therefore, using Luscinia was a convenience, but the same pattern was found with other software (both SAP and cross correlation).

Overall there are many missing details in how the data analyses were performed. I tried to flag that throughout the article with my line by line comments. Critical points are the justification of the choice for ranking syllables according to acoustic similarity instead of using a more linear metric (why not use directly the dissimilarity measure? mean rank could mean very different thing depending on where you are in the acoustic space)

- We wanted to have a fixed number of training stimuli in each of the two categories and that inherently involves ranking. Having done that, it made sense to similarly use ranking to select probes (but note that we used a very wide spread of ranking - between 20 and 200 from each of the training stimuli). The general point here is that we don’t think that this has affected the results in a meaningful way although one could choose a different approach.

and how bird individual variability is taken into account throughout the analyses.

- Although this is certainly an interesting point, studying individual variation in perception is beyond the scope of this paper. In order to maximise the variety of stimuli used different individuals were trained and tested with different stimuli.
- We only used birds with an accuracy of >65% over the training stimuli presented during that cycle (and 77% and higher for the evaluation set), we did not further analyse individual differences in accuracy.
- For the birds used in the model the mean accuracy of their decisions in response to training stimuli was 0.745 ± 0.062 (mean ± sd).

Furthermore, I think the paper would be strengthen by a thorough investigation of which acoustic features are important by zebra finch standards in the evaluation of syllable similarity. Revealing the perceptual map of birds would be a very interesting biological result.

- This is currently covered briefly by the "Song features" section (L440, figure S6). To go deeper than this correlation-based analysis would require a much larger number of data points, because of the very high dimensionality of the embedding space (needing, for coverage, a number of points exponential in the number of dimensions), and/or strong assumptions about the topology to be recovered.

Line by line comments

Line 50 « DTW » shouldn’t be « Luscinia »?

- Changed to *Luscinia* (L61)

138: which similarity measures were chosen? What heuristic is chosen for the ranking of syllables?

- We have now more clearly described this in L161: “*Using the DTW in Luscinia sound software (Lachlan, 2020) we compared all syllables with each other and extracted dissimilarity measures for each combination of syllables. Based on these dissimilarity measures, we subsequently constructed a matrix with, for each syllable, a ranking of all other syllables ranging from the most similar to the least similar syllable.”*

What prevent another bird from interfering with a given subject behavior when activating the operant feeder? Providing a picture of the design would give a better idea of the design.

- Each feeder was placed in a separate smaller, open cage within the aviary. This made it more difficult for birds to fly in to scrounge the activation of another bird. Nevertheless, it was never impossible for birds to either scrounge the reward of another bird, or to hop on a perch (and make a ‘choice’) after the activation of another bird’s stimulus playback. We have now more clearly explained this in L154-159: “*Since training and testing were done in a group setting birds could learn from, or scrounge from others' actions. Scrounging occurred either by displacing the original bird to claim the reward after a successful operation of the device, or by hopping on a choice perch after the focal bird triggered sound playback. Although this may have increased noise in our data, testing in a group setting made it possible to train and test continuously for months, without the welfare considerations that come with testing in social isolation.*”
- We have now also added a picture of the operant feeder in the appendix (Figure S1)

In figure S1, consider color coding the 2 sets of training stimuli A and B in the projection for clarity.

- We have now done so, colouring the left stimuli in red and the right stimuli in green, see figure S2.

What was the average distance of within category (7 most similar syllable to A) vs between categories (median(A) to median(B))?

- For the 99 stimulus sets the median dissimilarity within category is 0.06, whereas the median between category dissimilarity is 0.12.

Consider adding a figure that shows example spectrograms of one A-B set and a few probes with their Luscinia distances along with bird classification.

- We have now added figure S4 which shows these example spectrograms, the Luscinia dissimilarity and the bird’s classification. We refer to this figure in L400.

171: does 70% controls for bird ID = all birds have to be at 70% to move forward with probes?

- No, the group moved forward with probes when the group together have on average 70% accurate decisions. We have now phrased this more clearly: *After the group reaches on average 70\% accurate decisions with 8 stimuli on each side we added 10-15\% probes: L191.*

How often do bird not take decision (no perch hope/exit of the feeder)? How was such behavior quantified? Would it be counted as a wrong response?

- In this case we did not count cases where the bird did not make a decision as a wrong response. For the accuracy measurements and the probe responses we only counted trials in which a decision was made. In this group setting birds could easily be distracted after initiating a trial, and therefore it is nearly impossible to know whether no choice was made by mistake or because of the difficulty of the trial, or simply because the bird got interrupted or distracted. Therefore we did not count these trials without a choice.

175: what is the similarity metric used by Luscinia?

- We have removed this section, and replaced this analysis with an easier to interpret Figure 2.

175: on basis **of** their similarity

- This sentence has been removed.

176: the rank of ~~the~~ each probe stimulus

- This sentence has been removed

176: according to previous paragraph two sets of 8 stimuli are used with the probes. Here, is the initial (presumably close to the median?) stimulus of each set chosen as a reference for the triplet? How do you take into account the difference of variance within each set (e.g. differences across the 8 A stimuli)?

- Yes, the initial stimulus is chosen as reference for the triplet.
- The intra-8 variety is much lower than the inter-class variety. (See figures above)
- In designing the experiment we were limited by the fact that we did not know whether any particular acoustic method mapped closely to the birds perception.

177-179: rephrase this sentence because the sign of the difference totally depends on whether you are doing L-R or R-L, better indicate that the probe is more similar to the stimulus for which it has the smallest rank.

- We have now removed this section and replaced this analysis with an easier to interpret Figure 2.

180: over which stimuli is the mean rank difference calculated?

Consider adding mathematics formula to increase the transparency of calculations here.

- We have now removed this section and replaced this analysis with an easier to interpret Figure 2.

How many responses by the same bird to the same probe are obtained before assessing bird’s assessment? Do you merge all birds responses together?

- Just like for the previous figure 2, the new figure includes data from each assessment of birds with an accuracy of >65%.
- For this dataset, in total, birds assessed the same probe on average 3.93 ± 2.53 (mean ± sd). The minimum was 1, and the maximum was 16 times. We have now added this to L393.
- All these responses were merged for the figure.

What is a cycle of accuracy? How do you define a test period?

- Cycle accuracy was calculated as the proportion of correct responses among all responses to stimuli during the test phases. We have now added this to L189 and L325.
- We have now explained this more clearly in the caption of figure 1: *During pre-training birds were habituated to the device, and learned how to operate the device to obtain a food reward. After pre-training finished, they proceeded with their first testing cycle. Each testing cycle (step 4a – 5) consisted of a training period in which the birds were trained with new sets of stimuli (step 4a-4c) on each side, followed by a testing period (step 5) during which each bird is still presented with the same set of 8 training stimuli, but now interspersed by 10-15\% probes. Once a sufficient number of probes had been collected, the testing period, and with it the current cycle, was finished. The birds subsequently started a new cycle by starting training with a new stimulus set (step 4a).*

And in L195: *For each probe testing period, we aimed to collect approximately 1000 probe decisions in total (all birds together). After reaching this number the current cycle was finished and the birds would start a new cycle by starting training with a new stimulus set (see Figure 1).”*

192-194: nonsense, revise sentence.

- We have now changed this to: “*Our deep learning approach follows that of Morfi et al. (2021), but is adapted specifically to animal behavioural data, which can, due to the limited accuracy and consistency of measured animal judgments, contain a high proportion of uncertain (ambiguous) data points.”* We have now added this to L206.

222: what is the time window or bin size chosen for the mel spectrogram?

- The parameters for the Mel spectrogram computation are set as follows: the length of fast Fourier transform (FFT) window equals 2048, the number of samples between successive frames is set to 128 with window length 512. We have now added this information to L240.

234-235: what is defined as ambiguous and unambiguous behavioral data ?

- Ambiguous data contains a proportion of uncertain datapoints, in this case these are probes for which birds were not consistent in their assessments. Unambiguous data consists of datapoints without uncertainty, in this case where birds were consistent in their assessments. We have now explained this more clearly in L208: “*We define ambiguous data points to mean those for which, even after general data quality filtering, we do not have a consensus in the birds’ decisions for a given stimulus*.”

241: denotes the squared ~~the~~ Euclidean…

- Changed accordingly, and removed “squared” (L259).

177/240: it is somewhat confusing to choose a negative distance of rank to denote stimuli that are closer in the acoustic space according to Luscinia and then define positive stimuli (samples) for a given anchor as being closer than negative stimuli.

- We see that this might be confusing. These are two very different analyses, with different outcomes. However, because the figure and the analysis of assessing the birds assessments was so difficult to interpret we have now replaced it with a new Figure 2. We expect that this Figure is more easily to interpret and will not cause any confusion with the positive and negative stimuli relative to the anchor anymore.

242 and equation: define what N is (I assume total number of anchors = probes). A single triplet should only be a set of 3 stimuli and not the ensemble of triplets? According to the sentence, the Loss function is given for a single triplet, but the equation is giving it over all triplets in the dataset (sum over N?).

- We have now changed our sentence: “*For a triplet...of example stimuli” to “For dataset of N stimulus triplets...”* L260.

250: define cycle accuracy, explain what time binning is used to calculate it, what is the rolling time period?

- The cycle accuracy is explained in L189. We have now made this description clearer: “*Cycle accuracy is calculated as proportion of correct responses among all of a bird's responses to the training stimuli over the total of a cycle's test period.”*, and in L325: “*Cycle accuracy was calculated as the proportion of correct responses among all responses to stimuli during the test phases.”*
- This was calculated over the total probe test period for that cycle, which spanned several days. Since we aimed to collect approximately 1000 probe responses per cycle the length of the probe test period for a cycle, in days, depended on how often the birds interacted with the device on a day.

It’s an important point that scrounging is made possible in this design. An estimate of how often it happens would be important to develop here and present earlier when presenting the operant feeder.

- We have now added extra explanation about the possibility of scrounging to the section ‘Test paradigm’: *Since training and testing were done in a group setting birds could learn from, or scrounge from others' actions. Scrounging occurred either by displacing the original bird to claim the reward after a successful operation of the device, or by hopping on a choice perch after the focal bird triggered sound playback. Although this may have increased noise in our data, testing in a group setting made it possible to train and test continuously for months, without the welfare considerations that come with testing in social isolation. L154-159*
- In the current study we are not specifically interested in comparing the behavioural responses of individuals. Moreover, from the data collected it is impossible to deduct the incidences of scrounging or distinguish between decisions in which there was scrounging and decisions in which there was no scrounging. While supervising the birds we observed scrounging occurring; we estimate that this happened in less than 5% of the attempted trails.

What do you mean by “side decisions” do you mean systematic “errors”?

- With side decisions we mean that when presented more than once with a probe birds may not always be consistent in the side they choose, and thus with their similarity categorization. We have now changed this to “[…] *consistency of probes decisions caused by perceptual ambiguity of sounds.” L267*

261: what is a cycle?

- We have now explained this more clearly in the caption of figure 1: “*During pre-training birds were habituated to the device, and learned how to operate the device to obtain a food reward. After pre-training finished, they proceeded with their first testing cycle. Each testing cycle (step 4a – 5) consisted of a training period in which the birds were trained with new sets of stimuli (step 4a-4c) on each side, followed by a testing period (step 5) during which each bird is still presented with the same set of 8 training stimuli, but now interspersed by 10-15\% probes. Once enough probes had been collected, the testing period, and with it the current cycle, was finished. The birds subsequently started a new cycle by starting training with a new stimulus set (step 4a).”*

And in L195: “*For each probe testing period, we aimed to collect approximately 1000 probe decisions in total (all birds together). After reaching this number the current cycle was finished and the birds would start a new cycle by starting training with a new stimulus set (see Figure 1).”*

262: What was the representation of each syllable in these 2099 triplets? In other words in how many triplets of average was a given syllable being an anchor? Are some syllables found to be anchors more often than others?

- In total 547 probe syllables were used as an anchor in 2099 triplets. Each triplet was assessed on average 4.326 +/- 12.78 times (minimum 2, maximum 18). We have now added this information in L280.
- Each probe syllable was used on average in 3.81+/-2.91 triplets (minimum 1, maximum 18).

273-274: how is side consistency calculated, over how many number of responses to the same stimulus? Is it correct to assume like as on line 260-261 it is calculated for each bird? Perception choices could be highly dependent on individuals, how is that accounted for?

- Side consistency is calculated per individual per triplet, over at least 2 decisions. We have now clarified this a bit more in L277: *“[…] a consistent probe decision is one where, over at least two decisions, the same side was chosen for that probe during a cycle by an individual over 70\% of the times it was played to them*.”
- Perception choices can indeed be highly dependent on individuals. Since, in this study we are working with animals, who can, not only differ in their perception, but will also make mistakes in their decisions, we had to modify the deep learning method, as described in the paper.

Revise equation1 it is confusing with respect to the subsequent equations. Be consistent in the notations. Either D is the squared Euclidean distance or the Euclidean distance but not both.

- Our apologies, the word "squared" there appears as a typo: D refers to the Euclidean distance in eq 1, as elsewhere.

279-287: I am a bit confused by your new loss function in equation (4) as compared to (1). First the use of an exponential on the difference of distance for unambiguous tripets makes it highly susceptible to outliers: a single unambiguous triplet with a big difference of distance (pu>0) would very rapidly decrease the Loss to very low values (exponentially low!).
Note that Kumari et al (2019) does not use 1-exp(pu) but exp(-pu) for the unambiguous triplet. Please address why you are doing differently.

- The loss in eq 4 cannot be exponentially low for two reasons: first, this operates in a normalised embedding space (+-1 on each axis); thus for a worst-case triplet we might have 1-exp(2). Secondly, the overall loss function truncates the calculation by only keeping the positive part (the "[]+" surround, in eq4), so divergent low losses cannot occur. We tested various loss functions in pilot studies, including that of Kumari et al (2019) and Morfi et al (2021), finding the quoted loss function to be stable during training.

We have now added “*This loss differs slightly from that of Kumari et al (2019), and in pilot studies was found to be more stable for training*” to L308

Second, can you give an intuition for why adding a penalty to the difference of distance of ambiguous triplets is increasing the performance of distance prediction for unambiguous data?

- Our intuition is that the ambiguous decisions reflect a mixture of two things: experimental noise, and "genuine" ambiguity in that the triplet in question is not trivial to decide about. Put this together with the general fact that in deep learning, we need to constrain the search space as strongly as we can with the data we have got. Then, we arrive at the idea that we should teach the system that our ambiguous triplets should probably receive "ambiguous" embedding locations in the space - this can be interpreted as providing richer data to the training process, or alternatively as "regularising" the learning process. Either way, making proper use of the ambiguous data helps to train a better space, which yields better decisions even for the unambiguous triplets.

We have added “Deep learning benefits from diverse training data. The key intuition is that an ambiguous triplet is not bad data, but is inherently informative: the embedding should arrange points such that ambiguous triplets should not have an unambiguous embedding distance.” to L297

296: what do you call “Luscinia-U decisions in training”? Do you mean the ranking obtained through Luscinia acoustic measures of similarities? I see that this explain below. It is probably worth pointing to it or giving a short explanation here.

- We have now added “*Furthermore we explore models that use triplets created by Luscinia after tuning the algorithms parameters using the birds’ decisions (Luscinia-U triplets, as explained in more detail in the following section).”* to L311.

300-305: please spell out/give an equation for what you define as cycle accuracy, idem for the upper bound (self-rater agreement? Inter-rate agreement? How do you estimate these values?).

To explain cycle accuracy we have now added “...*calculated as the proportion of correct responses among all responses to stimuli during the test phases*.” in L325

- To explain the upper-bound we have added: *“[...] calculated as the percentage of decisions that agree with the majority decision for a given triplet, averaged over all triplets for which there was more than one decision recorded.”* in L327

Table 1: indicate how birds decisions are used in models checked for U and A: training + testing? How many triplets were constituting the Luscinia-U dataset (in other words by how much the bird dataset was augmented by these “machine” data?)?

- We have now added these numbers to the caption of Table 1: “*Luscinia triplets (L) refers to models that used Luscinia-U triplets for training (104457 triplets); unambiguous bird decisions (U) refers to models that used the triplet decisions created by the bird judgements through our operant experiments (2099 triplets: 1,239 training and 860 evaluation); ambiguous bird decisions (A) refers to models that used the triplet decisions created by the bird judgements through our operant experiments (1073 triplets: 668 training and 405 evaluation).”*

320: can you indicate how sound features are weighted in the original version of Luscinia for the calculation of the dissimilarity value, which I believe is the version used to rank syllables and choose the initial triplets with which birds were trained and tested? Note that it would be useful to describe this dissimilarity measure as a stand-alone paragraph earlier because it is used to decide of the behavioral sets of stimuli. Then the improvements in Luscinia U can go in a subsequent paragraph.

- The weightings in Luscinia used to measure the syllables with which the birds were trained were the same as the weightings in Table S1 (untrained), except for Peak frequency change and Wiener entropy which were omitted.

321: “same training data that was used to train neural networks” -> according to table 1 there are several versions of these data, can you be more specific? I assume you are using the behavioral data only, but then all triplets? Or only unambiguous triplets?

- We only used unambiguous triplets. We have now added this to L333.

322: how was the likelihood calculated for each triplet? The DTW algorithm described gives a single dissimilarity measure for each pair of stimuli, how did you convert these differences of distances into probability and loglikelihood?

- The relative dissimilarity between probe and the two training stimuli was calculated as: *X_p_=(D_probe-a_ – D_probe-b_)/( D_probe-a_ + D_probe-b_)*. The likelihood associated with that triplet was then expressed as: *1/(1+e^(4 X_p_)),* and we subsequently used a Metropolis-Hastings acceptance rule, with flat priors across potential parameter values to update or keep the weights in the used in the DTW algorithm. We have now added this information to L348: “*For each triplet in the dataset, we calculated the likelihood that the DTW algorithm would make the same choice as the birds, by calculating the dissimilarity between probe and the two training stimuli as: X_p_=(D_probe-a_ – D_probe-b_)/( D_probe-a_ + D_probe-b_). With this dissimilarity X _p_ we integrated log-likelihoods over the entire training dataset: 1/(1+e^(4 X_p_)), and used a Metropolis-Hastings acceptance rule, with flat priors across potential parameter values to update or keep the weights in the used in the DTW algorithm.”*

323: avoid jargon, “burn-in”

- We changed this to: “*[…] and discarded the first 1000 iterations*.” L354.

326: what was the rule in the MCMC approach to update or keep weights used in the DTW algorithm?

- We used a Metropolis-Hastings acceptance rule, with flat priors across potential parameter values (as described above).

335: dep->deep

- Changed accordingly

334: by “additional AXB triplet decisions” do you mean that you are adding triplets that have not been investigated by birds or that you are giving new responses to triplets for which you already obtained birds responses?

- These are triplets that have not been investigated by birds.

Figure S2: The schematic does not indicate how the bird behavioral responses are used to train the model. From the description in the paper we are expecting to see a model that predict the distances Dap and Dan from 3 sounds and this is not what is offered to the reader in this figure (well, the input seems to represent one spectrogram but we would expect three!). Then it would be useful to understand where the embedding projection is situated in the network (one but last layer?) relative to the actual final output which should be two values of distances.

Then what measure of distance are you computing from the embedding projections of two new input sounds to compare with other software dissimilarity value metrics?

- Figure S3 gives the neural network architecture which takes 1 spectrogram as input and outputs a single embedding location. Conceptually, the "triplet" aspect is not part of this model, but is part of the training process - the network shown in S3 is invoked 3 times, and then the 3 embedding locations are used to calculate the distances "Dap" and "Dan".

The measure of distance for comparing against others is simply the number of triplets for which Dap<Dan, which we could describe as the number of triplets whose positive/negative labelling is correctly classified in this representation.

We have (a) edited line 237, changing “*the deep learning model architecture*” to “*the deep learning model architecture for processing a single stimulus*” and (b) edited line 246-247, changing “*passed separately through the network depicted in Figure S3”*

350: an multiple -> a multiple

- Changed accordingly

353: was the Euclidean distance for each acoustic feature calculated with a weight of 1 for each of the 5 measurements (mean, max, min, start and end values) ?

- This is a mistake in the manuscript, as we only used the mean parameters. We have now changed this to: “*To test whether this happened, we first calculated, using Luscinia, for each syllable in the data-set, the syllable duration, as well as the mean of each of the following spectral acoustic features: fundamental frequency, peak frequency, median frequency, fundamental frequency change and harmonicity (Holveck et al., 2008).” (L375-378)*

362: given that the system was supposed to deliver 10-15% probe stimuli, how it comes that only 25,999 out of 1,116,214 trials were probe trials? Are you including in the count training phases (steps 1 to 4c?)

- Yes, in this total all trials in all cycles were included, from step 4a to step 5. We have now clarified this by adding “*(totals include all trials in the training phases step 4a-4c, and the testing phase step 5)*” in L391.

363: What does a testing period correspond to? how long were the testing periods? What was the decision criteria to switch from one period to the next?

- A testing period is the period during which we presented each bird with a set of 8 stimuli for each side and 10-15% probes. We aimed to collect approximately 1000 probes per group before switching to the next cycle. We have now clarified this in L194-196.

365: can you speculate why only half of the sets were discriminated above chance by the birds? Is there any correlation with the average acoustic distances (as evaluated by Luscinia?) within sets? Is the mean +/- SE the average value given over birds and the 46 sets? What does the SE represents here? Variability over birds and sets or birds only or sets only? Please provide a clear distribution of the cycle accuracy for all stim sets and all birds.

- Individual variation in training performance is very typical with operant conditioning. Here we have pioneered with a new method in which birds were housed in a group environment in the aviary, which is likely to have increased this variation in performance. There could be many reasons, (environmental, bird specific, task specific), why some birds did not discriminate >65% accuracy.
- There does not seem to be any correlation between the average acoustic distance and the cycle accuracy. See figure with the average dissimilarity between stimulus sets, and cycle accuracy below.
- We seem to have made an error in reporting the number of birds that performed >65% cycle accuracy. We have now corrected this: 59 birds, with an accuracy of 73.9% ± 5.5% (mean ± sd) in L396.
- We now report the standard deviation instead of the standard error.
- This variability is calculated over birds and sets.
- Please see below for the distribution of the cycle accuracies for the 99 stimulus sets.

Some more statistics of the dataset are missing to fully appreciate how representative the birds responses are. For instance: Were all 99 stimulus sets evaluated by all 23 birds? How many responses were obtained by a given bird for a given probe?

- Each stimulus set was used for training only one bird. We have now added this in L390.
- As described above: For the dataset used in the analysis, in total assessments of 547 probe syllables were used. Probes were assessed on average 4.326 +/- 12.78 times (minimum 2, maximum 18) by a given bird. We have now added this information in L280.
- Each probe syllable was used on average in 3.81+/-2.91 triplets (minimum 1, maximum 18).

369-371: I have a very hard time to understand what is represented in Figure 2 and how you are reporting the birds performance in the text. What is the standardized density of choices? Can you represent the birds’ response variability? Can you explain why the birds behavior is not symmetric with respect to zero? For some reason there seem to be a bias where distance to left stimuli needs to be smaller for being correctly classified as left stimuli as compared to right stimuli. Can you explain the peak at -0.2 for both left and right lines?

- The reviewer makes a good point that this is a very complex figure. We have therefore replaced this figure with a new, hopefully easier to understand, figure of the same data. “*To assess whether birds made the choices we expected, we compared the birds' assessments of similarity with assessments made by Luscinia. Dissimilarities between probe and stimuli, according to the Luscinia DTW algorithm, predicted zebra finch choices of perches (Figure 2).”*
- Please see Figure 2 in the manuscript, and the caption for the explanation.

Can you give any statistical test results for the comparison of model performances? How can you control for the different amount of data used?

- We used bootstrap estimation for the confidence intervals indicated in the error bars on Figures 3 and 4, for a simple unpaired verification of our main result. In deep learning literature, there is no standard practice approach to parametrise significance as a function of the training data set size - the effect of data set size would typically be explored empirically. Our data set is not large enough to make that exploration.
- Please note also that the training set size does not directly correspond to the number of training iterations for the algorithm, since we train using the standard deep learning practice of "early stopping" which adaptively determines the number of training iterations (details in Morfi et al 2021).

382-387: the method section explaining how the MDS projection is obtained is missing (what did you use as input to the MDS?....).

- The MDS projection in Figure 5 is not based on features but entirely on the AXB predictions/decisions made by the algorithms/birds. Its purpose is to illustrate which systems have similar decision tendencies as which others. We have expanded our description of the MDS in L414: “*This analysis does not use acoustic data but only the AXB predictions/decisions as input to MDS: the MDS distance between two algorithms should reflect the number of times they disagree*.”

The set of acoustic features used in Luscinia is not investigating temporal patterns of amplitude, that could account for the left over variations. Could you do a regression based on spectrogram distances to investigate what are the perceptual dimensions used by birds to perform this task? Alternatively it could be useful to investigate the embedding that best emulate birds behavior and interpret the maximum axis of variance: what are the birds paying attention to when they are doing this discrimination task on song syllables?

- It is possible that temporal variations in amplitude are responsible for some of the remaining variation, however, this can be a very difficult parameter to measure. Overall we would argue that song syllables are so highly multidimensional that simple summary statistics, such as the ones we examined are only likely to partially explain bird perceptual judgements. And, as argued above, we don’t think our data-set is best suited for a detailed exploration of this topic.

429-432: reference?

- We have now added a reference (L463).

451-453: I don’t see in the result section where you compared the acoustic parameters weights in U-Luscinia and the Betas of the model. Moreover, according to Table S1 and Figure S5 these acoustic features don’t seem to be exactly the same.

- This comparison was only a qualitative comparison. We have now edited the text to clarify this in L484: “*Moreover the relative contribution of different song features are similar to the parameter weight settings that resulted from tuning Luscinia with the birds' decisions (Table S1) in that fundamental frequency was found to be the most informative single feature.”*

**Reviewer #2:** Bird song learning is a major field of study both in behavioral biology and neurosience, in which it is used as a neural model system for human speech development (rodents and primates don't vocally imitate). Most studies depend on quantifying the level of learning that took place under experimental or natural conditions, in order to study how learning takes place. The level of resemblance between original song (tutor) and copy (tutee) is either scored by human observers or estimated by relatively simple algorithms. These algorithms use spectrographic features as input that human observers consider salient, and are thus indirectly also based on human judgement/iterpretation. By a combining two novel appoaches, the current study constitutes an qualitative advancement to this state of affairs, which has been the norm for decades.

First, the authors base the reference for similarity not on direct or indirect human judgement, but rather on the species own perceptual judgements. This makes a lot of sense because a tutee mimics a tutor's song through its own perceptual systems, and the level of success should be based on that. The authors introduce an innovative way to achieve this, namely testing a comparatively large number of birds psychoacoustically in an operant setup, while remaining in their normal social group setting. Traditionally, birds need to be isolated for such tests, which less ideal for social birds, as it may lead to stress and/or depression, which one should avoid as much as possible for ethical reasons, but which may also lead to inferior results. The results in the current study show that with the developed paradigm, it is possible to test comparatively large numbers of birds in a social situation. This will have important consequences as the the standard model species for speech development in neuroscience is the zebra finch, which is highly social. Until now most studies do not attempt to measure bird judgements, and if they do this is based on a very low number of subjects. Further, the authors show that the most commonly used similarity algorithms (human judgement based), do not closely predict bird perceptual judgement, although they do perform better than chance.

Second, the authors developed a deep-learning algorithm to predict the bird perceptual judgments. This algorithm outperforms both classic human judgement-based algorithms, as well a variant thereof that is weighted using the bird perceptual data. This shows that the novel deep-learning algorithm successfully includes features in the input data that are not captured with traditional techniques.

Taken together these are valuable results that undoubtedly will form the basis for better quantification of vocal similarity in many future studies.

However, I have some concerns and remaining questions after reading the manuscript:

1) How was it monitored that the birds received enough food? The only way the birds could get food is through the operant device (l.94). How was it verified that they were sufficiently successful? Presumably 50% correct is also a way to get food (since there is no real punishment), but it would be good to see that this was monitored in some way.

- We did three different ways: (1) Birds could feed ad libitum for 30 minutes at the start and at the end of each day, see L104, (2) Apart from multiple general welfare observations every day, also the performance of each bird, and the number of successful visits was monitored on a daily basis, and (3) birds were weighed before the experiment started and after 2 training cycles to check for unexpected changes in weight.
- We have now added the following text to the manuscript: “*Birds could feed ad libitum for a half hour after lights on, and a half hour before lights off. Operant feeders were operational from 7:30 to 18:30, which meant that the birds had to interact with the feeder to gain access to the food. During the pre-training period bird also received ad libitum access food for 15 minutes in the middle of the day. Every day the performance of each bird and its number of successful visits was monitored. Birds that did not interact with the apparatus or were consistently unsuccessful were removed from the experiment. This occurred in two cases: one bird did not interact with the device, and one bird developed an unrelated health problem during the experiment. Additionally, birds were weighed before the experiment started and after 2 training cycles to check for unhealthy changes in weight.” L104-L110*

2) The text is sloppy here and there in suggesting the neural network models the birds' sound perception (l. 247). The authors probably do not intend to literally claim this, but should be more precise. The model mimics the birds' categorization behavior based on the same sound input, but it could use correlated features.

- We have now changed this to: “*In order to build a perceptual space that reflects zebra finch perception, we require a set of easily distinguishable signals to avoid perceptual ambiguity.*” L265

3) A bit related to this: what is the actual input to the artifical neural network? The text mentions 'audio', which implies a sampled sound pressure waveform, but in l. 222 it is mentioned that recordings are first transformend into power mel spectrograms. This may be relevant because the transformation will perhaps filter out information that is normally available and used by neural auditory systems, such as phase. From the results it is clear that the algorithm does well without it, but it is relevant because such information could be a target for future improvements.

- We have now clarified this in L216 by adding “[…] *(transformed to power mel spectrograms),* […]”. We have also added more information about the transformation to power mel spectrograms in L238-243.
- The selection of log mel spectrogram as input was based on prior work which found these useful for deep learning applied to birdsong data, however, we don’t consider this to be the final word. This could be an interesting avenue for future research.

4) I can find little information on the likelihood of observational learning playing a role in the experiment. The birds can hear and see a bird respond in the operant aparatus I presume. Is this expected to have played a role?

- Birds could indeed hear and see other birds responding in the apparatus, and we believe that especially during the pre-training period this has helped with training them to trigger playbacks and to sit on the perches for access to the food reward. Once the actual training started each bird received its own set of training stimuli and from that moment onwards, listening to other birds’ stimuli would not benefit them in any way because they have their own, different, set of stimuli. There is the potential that observational learning could have affected their choices and introduced noise in the data. Also scrounging possibility could have affected their own learning performance. However we do not think listening to other birds’ stimuli would have affected their own classifications.
- We have now added an extra explanation about the possibility of scrounging and learning in the manuscript in L154-159.

l 101: Two speakers: why two and does this have an influence on the results? Design 1 had one speaker.

- We built design 2 to test a different paradigm, but in the end decided to use the test paradigm which we present in this manuscript. Both speakers played the exact same mono sound file simultaneously, so we do not expect any effects of this difference in design. We report this in the manuscript any way for the sake of completeness.

l. 119: "In quick succession". With an interstimulus interval? If so how long? Was the input to the algorithms also repeated? The end of one syllable and the start of the next one could be a 'feature' that is used by the birds.

- The interstimulus interval used between the playback of the stimuli was 20ms (now added to L134).
- The reviewer is correct to suggest that the end compared against the next start could be a strategy available to a listener. This is also true for sequences of syllables in natural zebra finch song. We judged that the interstimulus interval was large enough to reduce the strength of this possibility, though it remains possible in principle.
- The input to the algorithms was not repeated. Thus an algorithm would not be able to directly employ the strategy of comparing the end against the next beginning, but given that the whole syllable is available to the algorithm, it is in principle possible for the algorithm to make an analogous comparison if needed. For this, the dense layers at the end of the CNN would compare features from the beginning and end of the syllable spectrogram.

l. 165: Why is the criterion to move on to the next stage based on group performance? Why not per individual bird?

- This was based on the limitation of the computer code we designed. But for future studies it could certainly be interesting to do this based on each individuals’ performance.

l. 222: 170 time frames. The syllables had different durations I presume. Does that mean that the temporal resolution of time was different between syllables?

- Temporal resolution was the same in all syllables (details now added to text L238-243). Syllables shorter than our segment size were "zero-padded" with silence at the end.

l. 247: "model the way birds perceive sounds". I do not think this is necessarily what happens. The system categorizes zebra finch syllables like zebra finches do.

- *We have changed the sentence “In order to build a system that can model the way birds perceive sounds, we require a set of easily distinguishable signals to avoid perceptual ambiguity.*“ to “*In order to build a perceptual space that reflects zebra finch perception, we require a set of easily distinguishable signals to avoid perceptual ambiguity.” L265.*

l. 347: "syllable length", should perhaps be "syllable duration"?

- Changed accordingly
