# Supplementary material for: Bird song comparison using deep learning trained from avian perceptual judgments
Source: PLoS Comput Biol. 2024 Aug 7;20(8):e1012329. doi: 10.1371/journal.pcbi.1012329 (PMC11333001; doi:10.1371/journal.pcbi.1012329)
Supplement: Attachment — Submitted filename: Response_reviewer_comments.docx [file pcbi.1012329.s013.docx]

**Responses to reviewer comments**

Reviewer #1: With this revision, the authors have substantially increase their article clarity. Figure 2 is much more clear that the previous version. Overall the paper is really improved. My **general comments** are the following:

1- I think it is worth discussing the potential bias in the behavioral classification of syllables due to the choice of the training sets based on Luscinia's distance calculation. I appreciate the figure of pair distances (close/far) that the authors provided in their response, and understand that gathering new behavioral data is not reasonable, but I think it is important to discuss this aspect in the paper, and comment on how the change of distance metric could have affected the syllables ranking and choice of training sets. In the end, training a cohort of birds might not be much different than training an algorithm: whatever bias is present in the training set shows up in the algorithm/birds decisions.

- To be clearer about this we have added Figure S8 to the supplementary information and we have added the following paragraph to the discussion:

*“To train the birds to discriminate beyond two training syllable exemplars, we needed to ensure that they would generalise their decisions to other syllables too. To achieve this we trained the birds with two sets of 8 very similar syllables (rather than just two exemplar syllables). But this methodological choice created a potential conundrum: we had to make assessments of similarity between these 8 similar syllables using an existing method - in this case, Luscinia. Potentially, this might have influenced how birds learned: providing information about what was regarded as similar or not. At an extreme, training the birds might be the same as training them about the algorithm used to pick the training stimuli. Our protocol, however, involved selecting the 7 most similar syllables to an exemplar, with the rationale that the two categories of training stimuli would be very clearly separable and non-overlapping, no matter what the comparison method. We checked the similarity rankings of these training sets between Luscinia, cross-correlation and Sound Analysis Pro (Fig. S8), and in all cases the two training sets are very clearly separate, showing a higher similarity for the within-set syllables, than the between-set syllables. Therefore, we believe that the structure of our stimulus categories (for this part of the experiment) provides minimal information to the birds about the nature of similarity.” (L503-516)*

2- I think it would be useful to provide some contexts to, and work on the wordings of, the result sections line 434-448 (I tried to give some indications of what is unsettling below in my line by line comments).

- We have now worked on the wording of this section. Please see the responses to the line by line comments below.

3- Finally, it would worth it to investigate a bit more thoroughly the embedding obtained by the model (Song Feature section, which by the way, I would suggest to change the title of to something like: acoustic features used by the algorithm) and in particular increase the number of bioacoustic measurements used in the analysis. Interpreting the acoustic dimensions that the model is using to emulate the birds responses is valuable for understanding what birds might mostly value when evaluating song syllable similarity.

- We have now changed the title of the section in both the methods and the results to *Acoustic features used by the algorithm. (L376 and L456)*
- We have performed a more in depth analysis of the acoustic features to better understand which acoustic features best predicted the 64 dimension embedding of our algorithm. We have performed an analysis of 26 measures: 5 summary statistics (mean, maximum, minimum, start and end) for each of five acoustic features (fundamental frequency, peak frequency, fundamental frequency change, Wiener entropy, Harmonicity) plus syllable length. This analysis, accounted for 68% of overall dissimilarity between syllable embeddings, suggesting a wide range of predictor feature/statistic pairs. 13 of these pairs were significant and 8 of them had a standardised regression coefficient of >0.01. See results section L457-465.
- Additionally we have performed a second analysis where all summary measures are grouped by the 5 acoustic features and syllable length, and a third analysis examining the summary statistics start, end and mean. See results section L466-472.

Do you think birds might have adapted their perceptual judgement to the proposed set of training stimuli (weighting in more the acoustic features that were the most distinct between set A and set B of syllables) or are these results actually showing that they kept attending to features (such as the fundamental frequency) that were not necessarily the best to discriminate the two sets of syllables? Then it becomes interesting to see the importance of the fundamental frequency in emulating zebra finches' choices.

- Although I don’t think this is quite what the reviewer intended, we’d like to note that our analysis, which is based on the ML embedding, wouldn’t be suited to exploring individual differences between birds, or between training sets, since it is trained on the responses to all of them. More generally, we don’t think our data-set is suited to exploring the question of whether or not birds adjust their perception based on stimuli: the complexity of the stimuli combined with the selection of dissimilar training stimuli, means that all pairs of training stimuli were clearly distinctive in all underlying acoustic features. For example, we searched for the most similar pair of training stimuli according to their underlying fundamental frequency summary statistics, and they were still very clearly distinct in pitch, both to our ear and by measurement (e.g. the fundamental frequency over the first part of the syllable was 1700Hz on one and 1300Hz in the other). The pair of stimuli similarly clearly differed in the other acoustic features we can measure. By choosing a large set of stimuli pairs our intention here is that we provided our algorithm with training data that fairly reflects perceptual challenges faced by zebra finches in their natural behaviour.

**Line by line comments:**
L186-187 : Make clear that both stimuli are rewarded

- We have now clarified this: “In this training phase each bird is trained with its own set of stimuli: one stimulus is rewarded on the left side, and the other stimulus is rewarded on the right side. L186-187

Line 221: double point

- Removed the double point

L241: double parenthesis

- Parenthesis removed

L278-280: to be consistent, does a probe decision has to be the same over at least 2 decisions OR 70% of the times the probe was played to the birds? These two conditions do not seem to have the same contingencies to me, but maybe because on average a probe is presented 2-3 times, that ends up being 70%?. Also if it is a single individual response, why “played to THEM”?

- We have now changed this to: *a consistent probe decision is one where, a bird made at least two decisions for a particular probe during a cycle, and of those decisions, the bird chose the same side in >70% of the decisions. L279-281*

L326: Please add if I am correct, if not I am not sure to understand how the cycle accuracy is calculated: correct responses among all “training” responses to stimuli during the test phases.

- Yes, this is correct. We have now changed this accordingly: Cycle accuracy was calculated as the proportion of correct responses among all responses to training stimuli during the test phases. L328

L328: what is inter-rater here? Do you mean inter-bird?

- We have now changed this to: […] *based on the birds’ within- and between-bird agreement on the test probes* […] L330

L330-332: this sentence is awkward to me. I don’t quite make sense of the last part: “as estimated maximum accuracy (consistency assumption)”. Something seems to be missing.

- We have changed this sentence to: “*This is an optimistic upper bound since it relies on a consistency assumption (namely, that each bird's majority decision is the correct decision for a AXB triplet). We refer to it as the estimated maximum attainable accuracy (consistency assumption).” L332-334*

L353: do you mean “with flat priors across which parameter weights should be kept or updated in the DTW algorithm”?

- We have now changed this to: “*With this dissimilarity Xp we integrated log-likelihoods over the entire training dataset: 1/(1+e(4X p)), and used a Metropolis-Hastings acceptance rule, to update or keep the weights used in the DTW algorithm. We used flat priors across potential parameter values.” L353-355*

L352-355: Are they indeed 2 steps to decide of the weights (Metropolis-Hastings rule + MCMC) or is there some sort of repetition here that makes the text unclear?

- No this is indeed the same step. To make this clearer we have changed the text to: “*This* *Monte Carlo Markov Chain approach found DTW weightings that would maximise likelihood. The MCMC* […] L355-356

L393-395: can the authors indicate the number of different probe syllables for which data where obtained?

- The birds assessed 753 probe syllables all together. We have now added this: “Of these trials, 25,999 trials were probe trials from which we collected 22,048 sound assessments from the birds of in total 753 probe syllables.” L406-407

L432: revise something like: “We added all software methods to the MDS defined 2-d space that enables the comparison of model output.”

- Changed accordingly. L445-446

L434: rephrase “when evaluating systems”

- We have now rephrased this: “*When comparing software methods, we found that Sound Analysis Pro would yield similarities of precisely zero for some comparisons (in the case of high dissimilarity).*” L447

L434-435: this beginning of paragraph is unsettling: the reader has to guess what we are now looking at. “both pairs of similarities….” = algorithm similarity? Probe-training sound distances? Systems = algorithms/software methods?

- We have now changed this to: “*In triplets where the Sound Analysis Pro analysis yielded a zero value for the comparison between the probe and both training syllables of the triplet, we assume the three syllables to be so dissimilar to each other that no similarity judgement can be computed from Sound Analysis Pro; this was the case for 103 triplets.*” L448-451

L441 The transition to this paragraph is really dry. Consider adding some more context.

- We have now completely rewritten this section of the results to include the additional analyses. We now start the section with an introductory sentence:

*“We carried out 3 different MRM analyses to understand which acoustic features and summary statistics best predicted the 64 dimension embedding of our algorithm (Emb-LUA).” L457-458*

L442: According to methods L375-382 only 6 parameters are tested for their power to predict the distance matrix obtained with EmbLUA, where is the 26 acoustic features coming from?

- We have now replaced these results with new results for the additional analyses on acoustic features. L457-474

L445: I understand from these results that the embedding of EmbLUA can be explained at 56% by the differences in fundamental frequencies between song syllables, and then at 60.6-56 = 4.6% by the other 5 parameters tested, which leaves around 40% for other criteria. One parameter explaining 56% of the decisions criteria of Luscinia is not quite what I would name a comprehensive set of acoustic features… Maybe I am missing the point here, but the authors should revise the phrasing in this paragraph.

- We have now replaced these results with new results for the additional analyses on acoustic features. L457-474

FigS7: Please revise the legend. It is very difficult to make sense of it as is. E.g.: can the author specify here which model was tested (I presume EmbLUA?), what beta corresponds to on the yaxis (I guess weights of the regression model?). I can’t make sense of the legend sentence (“dissimilarity for the measures taken from fundamental frequency”?)

- We have now replaced these results with new results for the additional analyses on acoustic features. L457-474
- The new figure caption clarifies that the embedding is the Emb-LUA model, and that the y-axis shows the standardised regression coefficients from the MRM model (Fig 6).

L467-469: what would be the alternative? This task is a force choice test based on acoustic only, so I am not sure to understand what the authors mean here.

- The alternative would be that they pay no attention to the sounds or the acoustics characteristics of the sounds at all, but for instance, always choose a particular side of the apparatus, or choose randomly.
- To clarify this, we have now changed the sentence to: “*This suggests that overall the birds make decisions that correspond to acoustic characteristics, rather than, for instance, choosing randomly or always the same side*.” L494-495

L472-475: this sentence is hard to read, consider rephrasing in shorter sentences.

- We have changed this to: “*Taking into account, (1) our large sample size, (2) our inclusion only of birds that performed to a reasonably high level on training choices, and (3) evidence of agreement between trials when making the same choice on probes, we have made the assumption that a higher agreement between birds' choices and computational judgement reflects a greater similarity between bird perception and the algorithm.*” L498-502

471-472: The assumption made by the algorithm is also that the log mel spectrogram is a good representation of the zebra finch song syllables, which is not necessarily the case! When comparing the accuracy of the same classifier using different feature spaces (ad hoc bioacoustics features vs spectrogram vs mel scepstrum) at classifying zebra finches vocalization types, the mel space was found to be a poor feature space in Elie and Theunissen 2016. So it is possible that in addition to the explanations that the authors already mention, the input to the algorithm itself is not optimal. Could the author add examples of the actual input to the deep learning model ? Fig S4 is great in providing examples in the spectrogram space, but I would be curious to see the corresponding log mel spectrograms.

- We have now replaced the spectrogram images in Figure S4 with images of the actual mel spectrograms.
- Various representations have been compared in various bioacoustic machine learning projects (often Mel spectrograms, linear spectrograms, PCEN spectrograms), including some of our own, with no clear consensus that one type of spectrogram strongly outperforms another. We use a relatively large number of mel bands to ensure we do not have an overly-reductive input representation.

484-492: The authors make a strong statement that the birds and EmbLUA make use of spectro-temporal features that are not well represented by traditional bioacoustics measurements when they tested only 6 measures and one of them (fundamental frequency) actually explains alone more than half of EmbLUA embedding distances between syllables. I think this statement should be tuned down or results with a more comprehensive set of parameters should be provided.
Besides, this analysis is very interesting, because it opens a window into what acoustic features might be used by the birds.

- We have now expanded our set of acoustic features (25 measurements of acoustic features and syllable length, see comment above).
- We have now added the following text to the discussion: “*Zebra finch syllables are complex and variable. Within syllables, they vary from millisecond to millisecond in acoustic features including pitch, harmonicity and peak frequency (see fig. S4 for examples). When zebra finches are faced with a task of comparing two syllables, such as in our experiment or in their natural behaviour, they must integrate these differences. Our ML algorithm was trained on these decisions and it is therefore not surprising that the simple summary statistics of these features, such as means, only partially capture its embedding, together accounting for only 68% of variation.” L525-530*

To corroborate/illustrate the importance/usefulness for the birds of these acoustic features, one could look at the difference of them between unambiguous probe-training pairs of syllables and ambiguous probe-training pairs of syllables.

- *Adding an extra analysis on which probe-training pairs birds agreed on and on which pairs they did not, and how they differed in terms of acoustic features, would shift the paper markedly towards a paper on acoustic perception, rather than a paper on the assessment and development of computational methods of bird song comparison as we intended. We believe that this analysis would not give additional insights into those methods themselves.*

493: what is “our training dataset”? Name what you refer to with “new machine learning algorithm”? Is that EmbLUA? Since different datasets (bird generated, Luscinia generated) and different algorithms are presented in this paper, this sentence is a bit confusing.

- We have now changed this sentence to: “*The two algorithms that we trained with birds' judgments (Luscinia-U and our new machine learning algorithm Emb-LUA) outperformed other methods of song comparison on our testing data-set.” L538-539*
